# Fermentation of Orange Peels by Lactic Acid Bacteria: Impact on Phenolic Composition and Antioxidant Activity

**DOI:** 10.3390/foods13081212

**Published:** 2024-04-16

**Authors:** María del Carmen Razola-Díaz, Soumi De Montijo-Prieto, Eduardo Jesús Guerra-Hernández, María Jiménez-Valera, Alfonso Ruiz-Bravo, Ana María Gómez-Caravaca, Vito Verardo

**Affiliations:** 1 Department of Nutrition and Food Science, Campus of Cartuja, University of Granada, 18011 Granada, Spain; carmenrazola@ugr.es (M.d.C.R.-D.); ejguerra@ugr.es (E.J.G.-H.); 2 Institute of Nutrition and Food Technology ‘José Mataix’, Biomedical Research Center, University of Granada, Avda del Conocimiento sn, 18100 Granada, Spain; 3 Department of Microbiology, Campus of Cartuja, University of Granada, 18071 Granada, Spain; soumidemontijo@gmail.com (S.D.M.-P.); mjvalera@ugr.es (M.J.-V.); aruizbr@ugr.es (A.R.-B.); 4 Department of Analytical Chemistry, Faculty of Sciences, University of Granada, Avd. Fuentenueva s/n, 18071 Granada, Spain

**Keywords:** pomaces, HPLC-ESI-TOF-MS, submerged fermentation, food by-products

## Abstract

Orange processing generates peel by-products rich in phenolic compounds, particularly flavanones like hesperidin and narirutin, offering potential health benefits. Utilizing these by-products is of significant interest in supporting Spain’s circular bioeconomy. Therefore, the aim of this study was to investigate the fermentation of orange peels by different lactic acid bacteria (LAB) strains and its impact on phenolic composition and antioxidant activity. Three different LAB strains, two *Lactiplantibacillus plantarum*, and one *Levilactobacillus brevis* were utilized. The phenolic compounds were measured by HPLC-ESI-TOF-MS, and antioxidant activity was assessed using DPPH and ABTS methods. The growth of the LAB strains varied, showing initial increases followed by gradual declines, with strain-specific patterns observed. Medium acidification occurred during fermentation. A phenolic analysis revealed an 11% increase in phenolic acids in peels fermented by *La. plantarum* CECT 9567-C4 after 24 h, attributed to glycosylation by LAB enzymes. The flavonoid content exhibited diverse trends, with *Le. brevis* showing an 8% increase. The antioxidant assays demonstrated strain- and time-dependent variations. Positive correlations were found between antioxidant activity and total phenolic compounds. The results underscore the importance of bacterial selection and fermentation time for tailored phenolic composition and antioxidant activity in orange peel extracts. LAB fermentation, particularly with *La. plantarum* CECT 9567 and *Le. brevis*, holds promise for enhancing the recovery of phenolic compounds and augmenting antioxidant activity in orange peels, suggesting potential applications in food and beverage processing.

## 1. Introduction

In 2022/2023, the worldwide orange production amounted to 47.77 million metric tons, with orange juice production reaching 1.64 million metric tons [1]. The leading producers of orange juice in the year 2021/2022 were Brazil, the United States, and Mexico, with the European Union occupying the fourth position [1]. This means that around 3.8% of worldwide orange juice production originated from the European Union, amounting to 62,000 metric tons [1]. Moreover, Sapin is the main orange producer in the European Union, with oranges being the primary citrus fruit produced in the country [1]. Therefore, leveraging all aspects of orange production is of huge importance for the circular bioeconomy of the country.

During the processing of oranges to obtain their juice, by-products are generated, mainly consisting of the peel composed by the flavedo and albedo, a source of bioactive compounds. These compounds include phenolic acids, flavonoids, lignans, and anthocyanins [2]. Among the flavonoid phenolic family, especially the flavanones hesperidin and narirutin—hesperetin and naringenin metabolites, respectively—naturally present in the orange peel, have been attributed several bioactivities such as anti-inflammatory, antioxidant, anti-cancer, anti-diabetic, anti-rheumatic, and cardio-protective properties [3].

Fermentation is a fundamental biological process that has been harnessed by humans for millennia to transform raw materials into a wide array of valuable products, from food and beverages to pharmaceuticals and biofuels. This process involves the conversion of organic compounds, typically sugars or other carbohydrates, by microorganisms into various metabolites, such as alcohol, acids, or gases, often accompanied by the generation of energy. It plays a pivotal role in a broad range of applications, with profound implications for food production, industrial biotechnology, and environmental sustainability. The scientific understanding of fermentation has evolved over time, and, today, it encompasses a diverse array of microbial and enzymatic activities. Microorganisms, including bacteria, yeasts, and molds, are the primary agents responsible for carrying out fermentative processes [4]. Previously, orange peel has been submitted to solid-state fermentation by different Aspergillus species to produce citric acid (*Aspergillus niger*) [5,6], D-galacturonic acid (*Aspergillus oryzae* [7] and Aspergillus niger [8]), succinic acid (Fibrobacter succinogenes) [9], or other organic acids (*Aspergillus awamori*) [10]. Moreover, protein production from orange peel has been reported with *Aspergillus niger* and *Chaetomium* Spp. [11] and *Trichoderma reesei* and *Trichoderma viride* [12]. The production of industrially important volatile aroma esters from orange peels by yeast has been also reported [13]. Producing pigments using fungal strains (*Monascus purpureus* and *Penicillium purpurogenum*) from oranges’ processing waste has been reported by other authors [14]. Submerged fermentation by *Aspergillus fumigatus* for improving the content of ellagic acid in orange peels was reported by Sepúlveda et al. (2020) [15]. Ahmed et al. (2021) [16] optimized the pectinase of orange peel waste by *Penicillium chrysogenum* MF 318506.

Lactic acid bacteria (LAB) are a diverse and versatile group of Gram-positive microorganisms with a rich history of use in the food industry [17]. These bacteria play a pivotal role in food preservation, with their traditional role being the fermentation of carbohydrates to produce lactic acid at an industrial scale [18]. However, their significance extends far beyond this primary function. LAB have been found to produce a wide array of valuable by-products during their metabolic processes, including bacteriocins, vitamins, amines, short-chain fatty acids, and exopolysaccharides. Furthermore, the influence of LAB goes beyond their direct metabolic activities. When used in food fermentation, LAB have been shown to enhance the nutritional quality of the final products. In animal products such as milk [19] and meat [20], this enhancement is characterized by increased protein digestibility, improved mineral availability, and the release of peptides and amino acids, contributing to the overall nutritional value of the food [21,22]. Additionally, LAB can boost the antimicrobial and antioxidant properties of the fermented foods. In the context of specific food items, such as apple juice, mulberry juice, soy milk, and wheat dough, LAB-driven fermentation has been observed to elevate the levels of antioxidants and phenolic compounds [23,24]. LAB enzymes also play a key role in breaking down cell walls, which not only improves the release of phenolic acids and flavonoids but also converts them into simpler forms, enhancing their bioavailability [25]. This versatility makes LAB a cost-effective and sustainable technology for maintaining or improving the nutritional quality of food while preserving its sensory properties. The application of LAB in fruit and by-product fermentation can occur spontaneously in suitable conditions, thanks to the presence of naturally occurring lactic acid microbiota. Alternatively, it can be controlled and directed using specific lactic acid bacteria starter cultures to achieve various specific objectives, such as improved digestibility [26]. *Lactiplantibacillus plantarum* and *Levilactobacillus brevis* are LAB that share similarities as Gram-positive, facultative anaerobes, but they differ in several aspects [17]. *La. plantarum* exhibits greater acid and alcohol resistance, prefers warmer temperatures, produces a wide range of enzymes, and forms biofilms, whereas *Le. brevis* can grow at lower temperatures, has a more limited enzymatic capacity, and its biofilm-forming ability may vary. Although both species are part of the human intestinal microbiota and may have beneficial effects on health, they can interact differently with the immune system and intestinal epithelial cells. These interactions can influence the inflammatory response and the modulation of the immune system. Furthermore, different strains of *La. plantarum* and *Le. brevis* may vary in their susceptibility to antimicrobial agents. This is important in terms of food safety and the ability of these bacteria to compete with pathogenic microorganisms in fermented environments. Otherwise, genome sequencing has revealed a great genetic diversity within each species, which can influence their phenotypic characteristics and adaptability to different environments and substrates [27].

Despite their significant role in food science and nutrition, there remain intriguing avenues of research involving LAB, such as their potential to enhance the bioactivities of unique agro-industrial waste products, like orange peels. LAB can offer significant advantages in the valorization of food by-products. One key benefit is their remarkable ability to bioconvert the carbohydrates present in these by-products into valuable metabolites such as lactic acid and organic acids. This process not only stabilizes the pH of the substrate, prolonging its shelf life, but also transforms it into a more valuable resource for further processing. LAB fermentation can enhance the nutritional profile of food by-products by enriching them with bioactive compounds like polyphenols, vitamins, amino acids, and peptides, thereby increasing their overall nutritional value. Additionally, LAB-produced antimicrobial compounds can contribute to the preservation of these by-products, inhibiting the growth of spoilage microorganisms and pathogens. Furthermore, LAB fermentation could enhance the sensory characteristics of food by-products, imparting desirable flavors and aromas, which can enhance their market appeal. Overall, the utilization of LAB in the valorization of food by-products represents a sustainable approach that not only minimizes waste but also adds value to underutilized resources in the food industry [17].

LAB have been previously studied in orange peel with different purposes. Huang et al. (2017) [28] reported that orange peel fiber powder promotes growth, exopolysaccharide production, and antibacterial activity against the spoilage bacteria of *Lactobacillus plantarum* SLC 13, suggesting its potential for industrial applications as an economic prebiotic. In addition, de la Torre et al. (2020) [18] explored the use of *Lactobacillus delbrueckii sp. delbrueckii* for D-lactic acid production using orange peel as the substrate. Ricci et al. (2023) also studied the production of lactic acid from orange waste using a combination of *Aspergillus awamori* and *Lacticaseibacillus casei* and *Lacticaseibacillus rhamnosus* [29]. However, as far as we are concerned, LAB have still a wide range of applications in orange peels to be studied.

Therefore, the aim of this study was to develop industrially scalable biotechnological processes to revalorize and reuse orange peels. This was achieved through a combination of fermentation and ultrasound technologies for extracting bioactive compounds, especially phenolic acids and flavonoids.

## 2. Materials and Methods

### 2.1. Reagents and Samples

Gallic acid, DPPH, and ABTS were procured from Sigma-Aldrich (St. Louis, MO, USA). Water underwent purification through a Milli-Q system (Millipore, Bedford, MA, USA). Vanillic acid, chlorogenic acid, ferulic acid, quercetin, and rutin were likewise sourced from Sigma-Aldrich (St. Louis, MO, USA). HPLC-grade water and various other reagents were acquired from Merck KGaA (Darmstadt, Germany).

Orange by-products (variety Navelina) were secured after juice production. These by-products consisted of the albedo, flavedo, and the remaining pulp of the orange, with a moisture content of 70 ± 1.5%. For the experiments, the samples were subjected to drying at 60 °C with an air flow of 1.6 m/s for 315 min, achieving a moisture < 10%, as modeled and optimized previously by Razola-Díaz et al. (2023) [30]. The dried samples were grounded and stored in a frozen state at −18 °C until submitted to fermentation. The samples will be referred to as orange peels throughout the manuscript.

### 2.2. Lactic Acid Bacteria Strains and Culture Media

Lactic acid bacteria (LAB) strains were obtained from the Spanish Collection of Type Cultures (CECT): *Levilactobacillus brevis* CECT 5354, *Lactiplantibacillus plantarum* subsp. *plantarum* CECT 748T, and *Lactiplantibacillus plantarum* CECT 9567 (formerly, strain C4). The strains were grown in MRS medium at 26 °C for 24–48 h and preserved in glycerol stocks at −20 °C. *Le. brevis* CECT 5354 and *La. plantarum* CECT 748T were originally isolated from silage and pickled cabbage. In addition, *La. plantarum* CECT 9567 previously isolated from kefir was also used [31]. They all have demonstrated good results in previously published papers on the fermentation of avocado leaves and seeds [32,33].

### 2.3. Fermentation of Orange Peels

Orange peel fermentation with LAB strains was conducted in accordance with the protocols outlined by De Montijo-Prieto et al. (2023) [32]. Briefly, the bacteria were grown in MRS broth for 24 and 48 h at 26 °C, and the inocula were prepared in sterile saline and adjusted for each strain by turbidimetry, equivalent to a concentration between 10^7^ and 10^8^ colony-forming units (CFU)/mL. The viable bacterial counts in the inocula were determined by plating on MRS agar. The fermentation process involved the following steps: 1 g of orange peels was immersed in 8 mL of sterile water at 90 °C for 10 min to eliminate any microorganism present on the peels. After cooling, microbial counts were performed on Tryptic Soy agar (a nutrient-rich medium for a wide range of bacteria, incubated at 37 °C for 24–48 h), MacConkey agar (a selective medium for enterobacteria, incubated at 37 °C for 24 h), and Sabouraud agar (a medium for fungi, incubated at 30 °C for 7 days). Subsequently, the mixtures were inoculated with the previously prepared inocula to achieve a bacterial concentration ranging between 10^6^ and 10^7^ CFU/mL, followed by incubation at 26 °C. The CFU/mL were counted on MRS agar, and the pH values were measured and recorded after 24 and 48 h of incubation. A control group, prepared without the addition of lactic bacteria, was included in this study. The samples and control were each processed in duplicate. After incubation, both the samples and the control group were stored at −20 °C and subsequently freeze-dried for further analysis.

### 2.4. Extraction of Phenolic Compounds

The extraction of phenolic compounds from the fermented orange peels was carried out with an ultrasound sonotrode (UP400St ultrasonic processor, Hielscher, Germany) that works at 400 W and 24 kHz in the conditions previously optimized by Razola-Díaz et al. (2018) [2]. Briefly, 0.5 g of freeze-dried sample was subjected to extraction using 100 mL of ethanol–water at a ratio of 45:55 v/v and sonicated for 35 min at room temperature at an amplitude of 90% and a pulse 100%. Following this, the samples underwent centrifugation at 9840× *g* for 15 min. Subsequently, the supernatants were subjected to drying in a rotary evaporator under vacuum at 40 °C. The dried residue was then dissolved in 1 mL of methanol–water at a ratio of 50:50 v/v. The solutions were meticulously filtered through a 0.45 μm syringe filter and stored at −20 °C in amber bottles to prevent degradation until the time of analysis. It is important to note that each extraction was carried out in duplicate.

### 2.5. Determination of Phenolic Compounds by HPLC-ESI-TOF-MS

The extraction of the phenolic compounds in the orange peels fermented by selected LAB and a non-fermented control was analyzed in duplicate using the ACQUITY Ultra Performance LC system (Waters Corporation, Milford, MA, USA) linked to an electrospray ionization (ESI) source operating in a negative mode and a time-of-flight (TOF) mass detector (Waters Corporation, Milford, MA, USA). The desired compounds were separated on an ACQUITY UPLC BEH Shield RP18 column (1.7 µm, 2.1 mm × 100 mm; Waters Corporation, Milford, MA, USA) maintained at 40 °C, employing a gradient method previously described by Verni et al. [34]. This involved utilizing water with 1% acetic acid as mobile phase A and acetonitrile as mobile phase B. The obtained data were processed using the MassLynx 4.1 software (Waters Corporation, Milford, MA, USA). The compounds were identified by comparing the *m*/*z* and molecular formula with standards and previous research [2].

### 2.6. Antioxidant Assays: DPPH and ABTS

Antioxidant activity was assessed in all the samples using two distinct methods, and each method was performed in duplicate. The DPPH assay followed a procedure previously outlined by various researchers [35]. Specifically, 100 µL of each extract was combined with 2.9 mL of DPPH. The solution was vigorously stirred, and the fading of the extract’s color was monitored over a 30 min interval at 517 nm. The ABTS method, based on the method described by Re et al. (1999) [36], involved the generation of monocation ABTS^•+^ by oxidizing ABTS with potassium persulfate in darkness at room temperature for 12–24 h. For each extract, 1 mL of the ABTS solution was mixed with 0.01 mL of the extract, and the reduction in absorbance was recorded for 30 min at 734 nm.

## 3. Results and Discussion

### 3.1. Growth of LAB Strains in Orange Peels

As previously outlined, orange peels were supplemented with dextrose and yeast extract to encourage the growth of lactic acid bacteria (LAB). Viable microorganism counts were carried out after 24 and 48 h of incubation on MRS agar. The counts on MacConkey, Tryptic Soy, and Sabouraud agar confirmed that the heat treatment applied to the orange peels before lactic acid bacteria inoculation was effective in eliminating the microorganisms present on the peels, which could have interfered with the biotransformation of the phenolic compounds by lactic acid bacteria.

As indicated in Table 1, the orange peels demonstrated limited support for the growth of the LAB strains used for inoculation. The viable bacterial numbers increased in the first 24 h of incubation, persisting in a gradual decline throughout fermentation, except for *Le. Brevis,* which exhibited increased counts also after 48 h. Both *La. plantarum* strains achieved their peak growth after 24 h (8.18 ± 0.13 and 8.60 ± 0.03 log CFU/mL for strains CECT 748T and CECT 9567, respectively), while the exponential growth phase of *Le. brevis* was extended over 48 h, reaching comparable counts (8.21 ± 0.03 log CFU/mL). Our results are comparable to those obtained by Ricci et al. (2019) [37], who analyzed the growth of lactic acid bacteria on orange peel every 24 h for 5 days during a study which aimed to explore the feasibility of using orange peel as a raw material for the production of lactic acid by solid-state fermentation. Their results showed that *Lactiplantibacillus plantarum* strain 285 was able to grow well in the orange peels at different pH values (initial pH values of 5 and 6.5 and initial pH of 6.5 with CaCO_3_), increasing their cell number by 2 log cycles. This concentration remained unchanged during the subsequent days, without being affected by the pH. Regarding the pH values during the fermentation of the orange peels in our study, the growth of all the strains led to medium acidification (Table 1). After 24 h, the pH was reduced from 5.83 to 3.4 with *La. plantarum* CECT 9567 and to 3.7 with *La. plantarum* CECT 748T and *Le. brevis* CECT 5354. After 48 h, both pH values stabilized around 3.5.

### 3.2. Quantification of Phenolic Compounds by HPLC-ESI-TOF-MS and Biotransformation during Fermentation in Orange Peels

The orange peels fermented by the three strains for 24 and 48 h were submitted to extraction by sonotrode and analyzed by HPLC-ESI-TOF-MS.

As shown in Table 2, the major phenolic compounds in the orange peels were phenolic acids (hydroxycinnamic acids). Looking at the phenolic acid profile, there were no detected differences between the samples, and the phenolic acid profile was composed by 68% ferulic acid derivatives, 23% caffeic acid derivatives, and 9% sinapic acid derivatives. Phenolic acids have been previously reported to be the phenolic compounds most found in citrus peels, being ferulic acids the major one followed by caffeic acids [3], in agreement with the proportions found here. The bioactivities of these compounds have been extensively demonstrated [3]. In plants, hydroxycinnamic acids are found both esterified, covalently attached to the cell wall, and in soluble form [38,39]. The antibacterial effect of hydroxycinnamic acids, specifically in their action against lactobacilli, shows a relatively minor dependence on substitutions with hydroxyl or methoxy groups in the aromatic ring. However, it exhibits a pronounced reliance on the presence of a double bond in the side chain [40]. However, in our study, hydroxycinnamic acids, the phenolic group found in major concentrations in orange peels, were not in their aglycone form but in their conjugated form, which is much less toxic for LAB.

**Figure 1:**
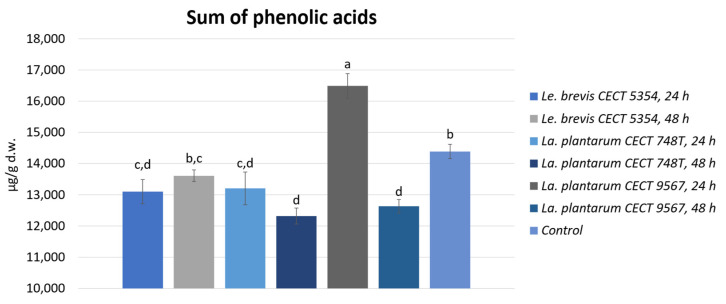
Sum of phenolic acids in fermented and non-fermented orange peels, expressed as the average and standard deviation. Different letters (a–d) indicate significant differences (*p* < 0.05).

As shown in Figure 1, looking to the sum of phenolic acids, a significant increment of 11% in the peels fermented by *La. plantarum* CECT 9567 can be seen after 24 h, with significant increments of 14.5, 6.8, and 14.4% for ferulic acid, caffeic acid, and sinapic acid derivatives, respectively, compared to the non-fermented orange peel. However, after 48 h of incubation, these hydroxycinnamic acid derivatives were decreased compared to the amounts found in the non-fermented orange peel. These differences when fermenting with *La. plantarum* CECT 9567 between 24 and 48 h of incubation could be related to biotransformation processes, such as the deglycosylation and hydrolysis of phenolic compounds, led by this bacterium during the incubation time. Phenolic compounds can be toxic to lactic acid bacteria. Even though *La. plantarum* has been widely studied for its adaptation to plant habits and capability to metabolize phenolics [41], it can be affected by some phenolic compounds via changes in the membrane’s fatty acid composition [42]. Thus, the phenolic compounds released during the incubation time could have had an antibacterial effect on the growth of this bacterium. In addition, the amount of 2-(E)-O-feruloyl-D-galactaric acid decreased significantly during fermentation with *La. plantarum* CECT 748T and *Le. brevis* CECT 5354 after 24 h of incubation.

Depending on the chemical structure and concentration of phenolic compounds, the growth and viability of lactic acid bacteria can be affected by affecting the cell wall and membrane [43]. Thus, lactic acid bacteria show several mechanisms for detoxification and tolerance to high concentrations of phenolics in plant niches high in phenolics for optimal growth and survival [43]. The metabolism of phenolic acids by lactobacilli, involving processes such as decarboxylation and/or reduction, is likely a predominant mechanism for detoxifying harmful compounds present in plant substrates encountered by lactobacilli [40]. The mentioned reductions in phenolic acids could be attributed to enzymes such as feruloyl esterases, which have been studied in many microorganisms, including lactic acid bacteria [38]. For example, the Est_1092 esterase from *La. plantarum* not only breaks down hydroxycinnamic esters (as a feruloyl esterase) and hydroxybenzoic esters (as a tannase) but is also a broad-range inducible esterase active on numerous esters of other phenolic acids [44]. Feruloyl esterases have also been studied for their activity in removing feruloyl residues that are esterified in pectin [32]. Since the pectin content in orange peel waste is between 20 and 40% [33], this enzyme could also de-esterify some residues as a part of the degradation process of the complex molecular structure of pectin together with polygalacturonase enzymes [34].

In contrast, the increment in some phenolic acid derivatives during fermentation with *La. plantarum* CECT 9567 could also be attributed to the glycosyltransferases that have been reported in lactic acid bacteria for their importance in exopolysaccharide production [45]. Glycosyltransferases (GTs) catalyze the formation of the glycosidic bond by transferring sugar moieties from donor molecules to a specific aglycon, with strict stereo/regioselectivities. The acceptors can be either sugars or other molecules such as polyphenols [46]. Due to the high availability of sugar naturally present in orange peels, it could activate the LAB production of exopolysaccharides. Previously, this action has been reported in some fungal strains [47,48,49]. Kralj et al. [50] reported, for the first time, the presence of a glucosyltransferase from a *Lactobacillus* strain. Thus, in this study, the increment in sinapic acid glucoside and glucuronide could be attributed to the action of the enzyme sinapate glucosyltransferase from sinapic acid, and the increase in caffeic acid glucuronide could be due to the action of the enzyme uridine-5′-diphosphate-glucuronosyltransferase from caffeic acid. This enzyme has been previously reported in Arabidopsis [51] and, a similar one, in *Bacillus subtilis* [52]. Also, some similar reactions have been reported in LAB. For example, a levansucrase from *Leuconostoc mesenteroides* B-512FMC can catalyze glycosyl transfer from sucrose to produce hydroquinone-β-fructoside [53]. A β-galactosidase from *Lactobacillus bulgaricus* L3 is able to catalyze galactosyl transfer, resulting in caffeic acid galactosidase [54]. The efficacy of phenolic acids’ glycosides is contingent upon the type of biological activity, the aglycone’s identity, and the specific identity and location of the glycone moiety. Observations from studies examining both glycosides and their corresponding aglycones suggest that phenolic glycosides generally serve as a storage or reserve pool for precursors of more bioactive compounds. Glycosylated compounds are likely to exhibit a higher bioavailability compared to their aglycone forms, attributed to the presence of sugar moieties. In the in vivo environment, hydrolysis of the glycoside would liberate the free aglycone, potentially enhancing its biological activity [55].

The flavonoids identified and quantified in fermented and non-fermented orange peels are presented in Table 3.

**Figure 2:**
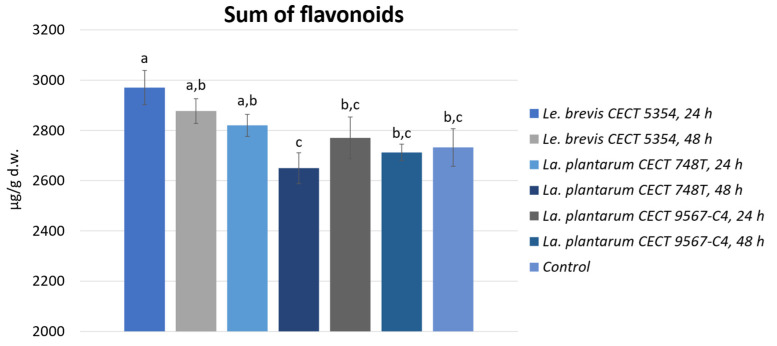
Sum of phenolic acids of fermented and non-fermented orange peels, expressed as the average with standard deviation. Different letters (a–c) indicate significant differences (*p* < 0.05).

Regarding the flavonoid profile, there were no detected significant differences between the samples, and the profile was composed by 33% hesperetin derivatives, 27% naringenin derivatives, 23% apigenin derivatives, and other minor compounds. Regarding the amounts, there were no significant differences in the case of the two *Le. plantarum* bacteria compared to the control, but *Le. brevis* was the most abundant, with a significative increment of 8% (Figure 2).

The major flavonoids detected were hesperidin, narirutin, and vicenin-2. Significant increments were observed mostly for the peels fermented by *Le. brevis* compared to the control: these increments were 11% for the sum of naringenin derivatives and 7.3% for the apigenin derivatives. Looking to the minor flavonoids quantified, that were quercetin derivatives, kaempferol derivatives, a luteolin derivative, and an isorhamnetin derivative; the tendency was similar, with the best results for *La. plantarum* C4 and *Le. brevis* measured after 24 h. There was a clear tendency of reducing the flavonoid content after 48 h for all three of the tested bacteria compared to 24 h of fermentation. The significant increase obtained could be attributed to the synergistic effect between fermentation and ultrasound extraction. Moreover, the increments in prunin, narirutin, and hesperidin could be due to the individual or combines action of the enzymes flavanone-7-O-glucosyltrasnferase and rahmnosyltransferase from the aglycones forms of hesperetin and naringenin [56]. Among the lactobacilli genera, *La. plantarum* and *Le. brevis* have been reported to have the capability to metabolize these two citrus flavanones [57]. Regarding the other minor flavonoids, the mechanisms have also been similarly reported [32,33].

No previous references were found regarding the fermentation of orange peel, focusing on its effect on the peel’s phenolic composition. Just recently, Deba-Rementeria et al. (2023) [58] proved the acceptability of fermented orange peel-based beverage and snacks, fermented using *La. plantarum* CECT 749 for 10 days, with the addition of sucrose with 2% NaCl. However, they had a significative reduction of about 50% in the total flavonoid contents but with improvements in the color of the fermented orange peels, which seemed to be more luminous, having a higher color intensity and more yellowness, probably associated with a greater transformation of the peels because of higher microbial activity during the process, with a higher lactic acid content and a less bitter taste than beverages and snacks created from non-fermented orange peels.

Thus, the selection of the fermenting bacteria and fermenting time for orange peels is a determining factor and should be based on the final purpose of the extract.

### 3.3. Antioxidant Activity in Fermented Orange Peel

**Figure 3:**
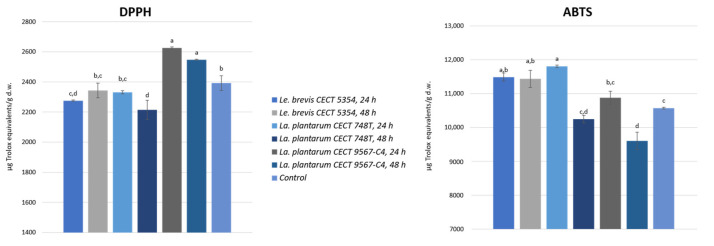
Antioxidant activity of fermented orange peels, using the three different strains, after 24 and 48 h and a control, expressed as the average with the standard deviation. Different letters (a–d) for each method indicate significant differences (*p* < 0.05) between the samples.

DPPH and ABTS antioxidant assays were performed on the fermented samples and a control, and the obtained results are presented in Figure 3.

The results obtained ranged from 2214.4 to 2625.0 µg TE/g d.w. for DPPH and from 9604.9 to 11,807.8 µg TE/g d.w. for ABTS. These results are in the same range of magnitude as previous research [30,59]. It is a fact that the polar fractions containing phenolics and flavonoids from lime, lemon, sweet orange, and grapefruit peels have been extensively reported to display a greater antioxidant potential than the volatile fractions containing essential oils from the peels [3].

As it can be seen, different tendencies were obtained, but with significant increments compared to the control for some bacteria. In the two *La. plantarum* strains tested, there were reductions after 48 h compared to 24 h of fermentation, but, for *Le. Brevis,* it seemed to be the opposite. For the DPPH assay, the orange peel fermented using *La. plantarum* CECT 9657, after 24 or 48 h, reported the highest results, with a significant (*p* < 0.05) increment compared to the control. Moreover, the orange peel fermented using *Le. Brevis,* after 48 h, or the other *La. plantarum* strain, after 24 h, did not present significant reductions in terms of its antioxidant activity in comparison with the non-fermented peel. In the ABTS assay, orange peel fermented using the three tested bacteria exhibited a higher antioxidant activity than the control. Among them, the orange peel fermented using *Le. brevis* (both after 24 and 48 h) and *La. plantarum* CECT 748T (after 24 h) had the highest radical scavenging activity against ABTS cations. Some authors reported increases in antioxidant activity when fermenting avocado seeds [33,60] and apple juice [61] with *La. plantarum*. Also, a higher antioxidant activity in litchi juice [62] and mulberry juice [63] was found when fermenting them with *Le. brevis.*

**Figure 4:**
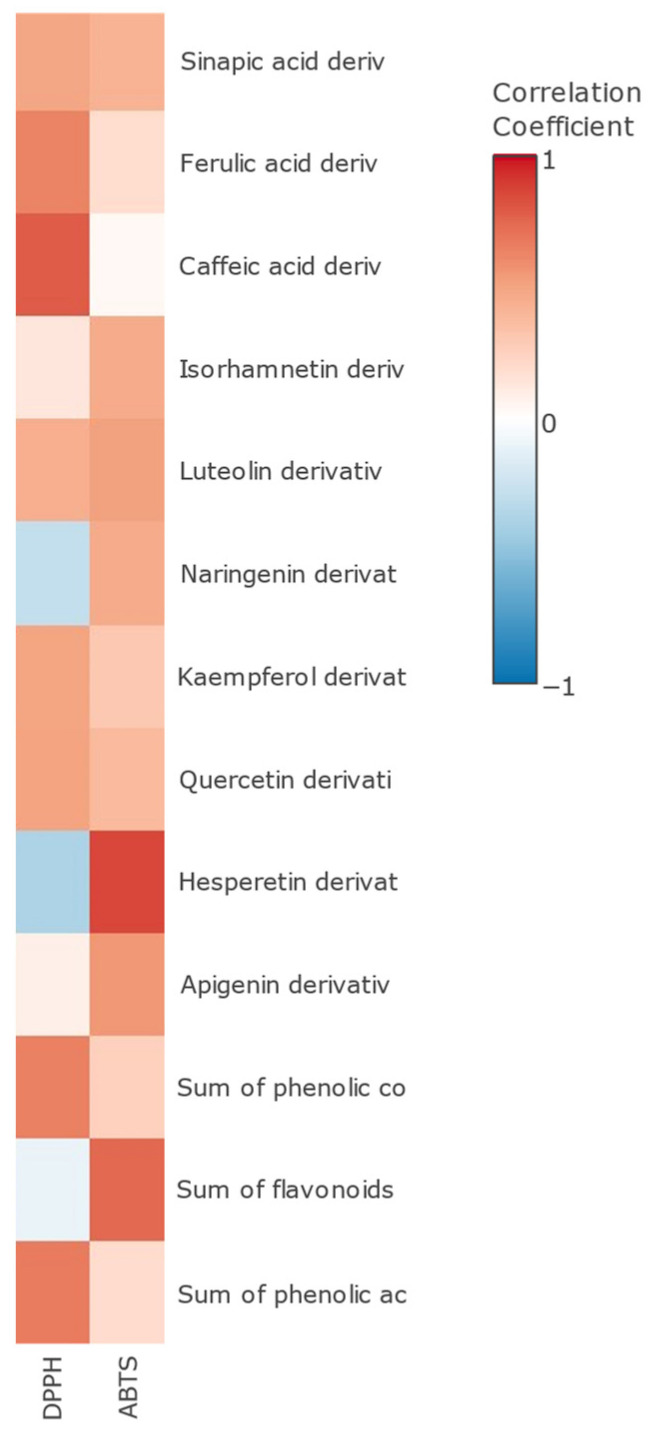
Pearson’s correlation heatmap of antioxidant activity by DPPH and ABTS and phenolic compounds by groups.

Furthermore, a Pearson’s correlation test was performed (Figure 4). Both methods of analysis showed a significant positive correlation with the sum of total phenolic compounds. DPPH had a higher correlation with the sum of phenolic acids (r = 0.7861, *p* < 0.05), especially caffeic acid derivatives (r = 0.8056, *p* < 0.05). As previously noted, caffeic acid-O-glucuronide exhibits antioxidant activity comparable to its parent compound, caffeic acid. The significance of a 4′-hydroxyl group on the aromatic ring appears to be pivotal for its antioxidant activity, particularly in the context of the DPPH assay [64]. ABTS had a higher correlation with the sum of flavonoids (r = 0.7725, *p* < 0.05) and especially with hesperidin (r = 0.8943, *p* < 0.05). Previously, Abdallah et al. reported a higher correlation between hesperidin and ABTS than with a DPPH antioxidant assay in orange by-products [65].

## 4. Conclusions

This study investigated the impact of LAB fermentation on orange peels’ phenolic composition and antioxidant properties. While the orange peels showed limited support for LAB growth, the different strains exhibited varied growth patterns and acidification during fermentation. The phenolic acids increased by 11% in the peels fermented using *La. plantarum* CECT 9567 after 24 h, possibly due to glycosylation by LAB enzymes. The flavonoid content remained stable, except for an 8% increase in the peels fermented using *Le. brevis*. The DPPH and ABTS antioxidant assays revealed significant activity increments, particularly with *La. plantarum* CECT 9567 after 24 h and *Le. brevis* after 48 h. Antioxidant activity correlated positively with the total phenolic compounds. These findings underscore the importance of selecting appropriate bacteria and fermentation durations to tailor phenolic composition and antioxidant activity, suggesting that LAB fermentation enhances phenolic compounds’ recovery by up to 20%. These results emphasize LAB fermentation’s potential to enhance orange peel applications in food and beverage processing.

## Figures and Tables

**Table 1:** Log CFU/mL and pH of lactic acid bacteria in orange peels expressed with average ± standard deviation.

	0 h		24 h		48 h	
	Log10 (CFU/mL)	pH	Log10 (CFU/mL)	pH	Log10 (CFU/mL)	pH
*Le. brevis* CECT 5354	7.92 ± 0.03	5.8	8.09 ± 0.09	3.7	8.21 ± 0.03	3.5
*La. plantarum* CECT 748T	7.94 ± 0.02	5.8	8.18 ± 0.13	3.7	8.01 ± 0.02	3.5
*La. plantarum* CECT 9567	7.90 ± 0.03	5.8	8.60 ± 0.03	3.4	8.51 ± 0.01	3.2

**Table 2:** Phenolic acids quantified in the fermented orange peels and a control by HPLC-ESI-TOF-MS, expressed as the average ± standard deviation in µg/g d.w.

Compound	*Le. brevis* CECT 5354		*La. plantarum* CECT 748T		*La. plantarum* CECT 9567		Control
	24 h	48 h	24 h	48 h	24 h	48 h	
Caffeic acid 3-O-glucuronide isomer I	247.31 ± 6.16 ^b^	292.08 ± 4.35 ^c^	299.02 ± 8.16 ^b^	291.53 ± 7.42 ^b^	344.24 ± 10.58 ^a^	331.17 ± 10.82 ^a^	324.25 ± 2.11 ^a^
Caffeic acid 3-O-glucuronide isomer II	1013.41 ± 9.26 ^b^	1086.94 ± 2.52 ^a^	962.02 ± 35.99 ^c^	926.84 ± 9.88 ^c^	1136.69 ± 9.70 ^a^	835.58 ± 1.85 ^d^	1031.70 ± 26.31 ^b^
Caffeoylglycolic acid methyl ester	566.91 ± 4.51 ^d^	586.10 ± 0.43 ^d^	699.64 ± 32.07 ^a^	609.64 ± 6.84 ^c,d^	658.41 ± 20.12 ^a,b^	672.53 ± 11.32 ^a,b^	643.56 ± 4.80 ^b,c^
Caffeoylmalic acid isomer I	784.93 ± 16.56 ^c^	822.56 ± 21.66 ^b,c^	913.38 ± 32.92 ^a^	816.35 ± 12.40 ^b,c^	925.70 ± 33.03 ^a^	939.33 ± 12.71 ^a^	878.32 ± 20.35 ^a,b^
Caffeoylmalic acid isomer II	358.13 ± 10.94 ^c^	365.61 ± 11.82 ^c^	432.31 ± 20.23 ^a,b^	362.31 ± 4.25 ^c^	460.45 ± 9.25 ^a^	440.02 ± 10.83 ^a,b^	426.22 ± 4.97 ^b^
2-(E)-O-Feruloyl-D-galactaric acid isomer I	535.18 ± 25.98 ^e^	618.73 ± 3.04 ^c^	602.65 ± 25.53 ^c,d^	561.19 ± 7.69 ^d,e^	785.52 ± 19.31 ^a^	582.45 ± 27.15 ^c–e^	682.90 ± 15.40 ^b^
2-(E)-O-Feruloyl-D-galactaric acid isomer II	959.38 ± 30.16 ^c,d^	1013.35 ± 9.56 ^b,c^	923.94 ± 45.24 ^d^	907.83 ± 17.36 ^d^	1217.10 ± 37.75 ^a^	890.69 ± 8.30 ^d^	1081.95 ± 11.02 ^b^
2-(E)-O-Feruloyl-D-galactaric acid isomer III	1114.06 ± 12.43 ^c,d^	1176.40 ± 48.97 ^b,c^	1040.48 ± 50.14 ^d,e^	993.00 ± 42.88 ^e,f^	1578.57 ± 47.40 ^a^	913.48 ± 24.26 ^f^	1253.86 ± 9.81 ^b^
2-(E)-O-Feruloyl-D-galactaric acid isomer IV	2660.23 ± 110.54 ^c^	2834.30 ± 2.67 ^b^	2447.51 ± 88.72 ^d^	2370.97 ± 22.84 ^d,e^	3395.69 ± 25.11 ^a^	2261.85 ± 46.95 ^e^	2882.53 ± 33.35 ^b^
2-(E)-O-Feruloyl-D-galactaric acid isomer V	1435.76 ± 67.27 ^c,d^	1503.91 ± 32.86 ^b,c^	1388.00 ± 49.60 ^c,d^	1307.67 ± 31.65 ^d^	1984.38 ± 90.00 ^a^	1288.17 ± 11.13 ^d^	1603.01 ± 65.47 ^b^
Feruloyl isocitric acid isomer I	322.02 ± 15.04 ^c,d^	309.86 ± 12.00 ^d^	342.57 ± 13.62 ^b,c^	313.79 ± 8.48 ^c,d^	403.49 ± 5.95 ^a^	391.67 ± 8.09 ^a^	354.75 ± 4.66 ^b^
Feruloyl isocitric acid isomer II	1122.62 ± 24.61 ^c,d^	1112.85 ± 8.54 ^c,d^	1187.72 ± 53.48 ^b,c^	1102.26 ± 32.63 ^d^	1383.98 ± 34.79 ^a^	1361.08 ± 1.97 ^a^	1232.35 ± 9.59 ^b^
Feruloyl isocitric acid isomer III	485.96 ± 9.92 ^b,c^	450.20 ± 2.54 ^c^	535.49 ± 19.92 ^a^	466.59 ± 15.53 ^c^	536.72 ± 16.04 ^a^	512.50 ± 10.84 ^a,b^	507.51 ± 6.34 ^a,b^
Ferulic acid O-glucoside	302.87 ± 5.65 ^a^	277.48 ± 1.75 ^b^	259.58 ± 2.74 ^b,c^	253.43 ± 6.73 ^c,d^	240.32 ± 9.06 ^d^	194.82 ± 6.28 ^e^	252.46 ± 9.04 ^c,d^
Sinapic acid O-glucoside	241.00 ± 6.57 ^a^	224.12 ± 0.18 ^b^	215.36 ± 2.51 ^b,c^	203.23 ± 0.98 ^c^	211.73 ± 6.59 ^b,c^	142.59 ± 5.61 ^d^	211.80 ± 4.18 ^b,c^
Sinapinic acid-O-glucuronide isomer I	811.12 ± 27.33 ^c^	805.08 ± 18.41 ^c^	795.88 ± 34.20 ^c^	703.32 ± 23.89 ^d^	1070.21 ± 14.86 ^a^	726.92 ± 17.21 ^d^	873.06 ± 0.10 ^b^
Sinapinic acid-O-glucuronide isomer II	138.55 ± 5.43 ^b,c^	130.91 ± 5.17 ^c^	158.04 ± 7.84 ^a^	129.10 ± 3.20 ^c^	154.65 ± 6.99 ^a^	145.25 ± 0.07 ^a,b^	147.95 ± 1.46 ^a,b^

Different letters (a–f) in the same line indicate significant differences (*p* < 0.05).

**Table 3:** Flavonoids quantified in the fermented orange peels and a control by HPLC-ESI-TOF-MS, expressed as the average ± standard deviation in µg/g d.w.

Compound	*Le. brevis* CECT 5354		*La. plantarum* CECT 748T		*La. plantarum* CECT 9567		Control
	24 h	48 h	24 h	48 h	24 h	48 h	
Quercetin-3-O-rutinoside-7-O-Glucoside	27.64 ± 1.17 ^a,b^	25.46 ± 0.88 ^c,d^	25.44 ± 0.77 ^c,d^	23.84 ± 0.76 ^d^	28.64 ± 0.72 ^a^	23.97 ± 0.05 ^d^	26.23 ± 0.20 ^b,c^
Luteolin 7-O-glucoside	62.27 ± 0.95 ^a,b^	61.33 ± 2.26 ^a,b^	57.68 ± 1.99 ^a,b^	52.04 ± 1.93 ^c^	62.68 ± 1.65 ^a^	57.29 ± 2.35 ^b^	59.21 ± 0.77 ^a,b^
Prunin	149.44 ± 1.35 ^a^	141.97 ± 2.65 ^b^	138.38 ± 2.75 ^b^	120.88 ± 1.30 ^c^	124.48 ± 1.71 ^c^	137.31 ± 0.14 ^b^	123.74 ± 2.72 ^c^
Isorhamnetin-3-O-rutinoside isomer I	95.29 ± 3.24 ^a^	91.70 ± 0.63 ^a,b^	85.68 ± 2.65 ^c^	88.01 ± 0.35 ^b,c^	95.80 ± 1.46 ^a^	84.86 ± 1.59 ^c^	92.54 ± 1.41 ^a,b^
Isorhamnetin-3-O-rutinoside isomer II	45.12 ± 2.14 ^a^	43.67 ± 1.40 ^a,b^	40.22 ± 1.72 ^b,c^	38.48 ± 0.79 ^c^	43.74 ± 1.51 ^a,b^	38.71 ± 1.74 ^c^	42.46 ± 1.14 ^a–c^
Vitexin-O-pentoside	128.65 ± 0.81 ^a^	125.94 ± 0.01 ^a,b^	121.50 ± 3.51 ^b,c^	116.85 ± 0.21 ^c^	125.34 ± 4.05 ^a,b^	121.79 ± 0.52 ^b,c^	122.45 ± 0.45 ^b,c^
Naringin hydrate	18.83 ± 0.48 ^b,c^	19.03 ± 0.90 ^a-c^	17.68 ± 0.68 ^c,d^	16.71 ± 0.81 ^d^	20.67 ± 0.72 ^a^	16.97 ± 0.41 ^d^	19.49 ± 0.06 ^a,b^
Apigenin-di-C-hexoside (Vicenin-2) isomer I	490.70 ± 6.40 ^a^	481.67 ± 0.88 ^a^	473.24 ± 9.59 ^a,b^	431.92 ± 3.21 ^c^	448.12 ± 15.82 ^b,c^	470.90 ± 1.79 ^a,b^	444.48 ± 19.28 ^b,c^
Apigenin-di-C-hexoside (Vicenin-2) isomer II	28.47 ± 0.89 ^b^	27.58 ± 0.02 ^b^	27.71 ± 1.17 ^b^	28.48 ± 0.84 ^b^	32.04 ± 0.79 ^a^	27.40 ± 0.91 ^b^	30.84 ± 0.35 ^a^
Apigenin-di-C-hexoside (Vicenin-2) isomer III	17.27 ± 0.72 ^d^	17.88 ± 0.49 ^c,d^	17.30 ± 0.02 ^d^	18.77 ± 0.48 ^b,c^	21.41 ± 0.30 ^a^	17.44 ± 0.75 ^c,d^	20.03 ± 0.49 ^a,b^
Apigenin 7-O-neohesperidoside	28.46 ± 0.58 ^a^	27.64 ± 0.78 ^a^	26.48 ± 0.96 ^a–c^	23.68 ± 0.67 ^d^	27.31 ± 0.99 ^a,b^	24.98 ± 0.57 ^c,d^	25.38 ± 0.10 ^b–d^
Narirutin	621.47 ± 8.42 ^a^	610.59 ± 5.64 ^a,b^	585.25 ± 2.80 ^b,c^	558.30 ± 15.65 ^c,d^	537.37 ± 18.90 ^d^	588.76 ± 12.61 ^a–c^	560.05 ± 16.29 ^c,d^
Rutin isomer I	97.43 ± 2.67 ^a,b^	92.69 ± 2.28 ^b,c^	86.39 ± 2.88 ^c,d^	84.59 ± 3.54 ^d^	100.77 ± 0.51 ^a^	89.37 ± 0.85 ^c,d^	91.25 ± 3.82 ^b–d^
Rutin isomer II	63.38 ± 2.13 ^a,b^	63.58 ± 2.20 ^a,b^	60.96 ± 2.48 ^b^	57.69 ± 1.75 ^b^	68.30 ± 3.12 ^a^	58.31 ± 1.47 ^b^	63.48 ± 1.21 ^a,b^
Hesperidin	960.16 ± 31.76 ^a^	907.31 ± 23.27 ^a,b^	924.52 ± 5.57 ^a,b^	863.24 ± 25.80 ^b,c^	873.10 ± 26.05 ^b,c^	833.32 ± 4.76 ^c^	868.09 ± 23.69 ^b,c^
Neohesperidin	17.48 ± 0.12 ^b,c^	18.85 ± 0.59 ^a^	17.79 ± 0.36 ^a,b^	15.84 ± 0.03 ^d^	16.24 ± 0.12 ^d^	16.51 ± 0.48 ^c,d^	16.09 ± 0.79 ^d^
Alpha-Glucosyl Hesperidin isomer I	<LOQ	<LOQ	<LOQ	<LOQ	<LOQ	<LOQ	<LOQ
Alpha-Glucosyl Hesperidin isomer II	<LOQ	<LOQ	<LOQ	<LOQ	<LOQ	<LOQ	<LOQ
Kaempferol 3-O-[3″,6″-di-O-(E)-cinnamoyl]-b-D-glucopyranoside isomer I	14.98 ± 0.60 ^b,c^	16.14 ± 0.66 ^a,b^	15.62 ± 0.72 ^b,c^	14.80 ± 0.56 ^b,c^	17.41 ± 0.87 ^a^	14.41 ± 0.15 ^c^	16.00 ± 0.06 ^a–c^
Kaempferol 3-O-[3″,6″-di-O-(E)-cinnamoyl]-b-D glucopyranoside isomer II	19.89 ± 0.57 ^a,b^	20.46 ± 0.52 ^a^	19.45 ± 0.83 ^a–c^	17.98 ± 0.70 ^c^	20.54 ± 0.90 ^a^	18.36 ± 0.52 ^b,c^	19.82 ± 0.05 ^a,b^
Kaempferol 3-[2″-glucosyl-6″-acetyl-galactoside]7-glucoside isomer I	28.58 ± 1.10 ^c,d^	29.77 ± 0.75 ^b,c^	28.32 ± 0.89 ^c,d^	27.95 ± 0.69 ^c,d^	33.89 ± 1.22 ^a^	26.92 ± 0.20 ^d^	31.60 ± 0.10 ^b^
Kaempferol 3-[2″-glucosyl-6″-acetyl-galactoside]7-glucoside isomer II	31.98 ± 1.25 ^b^	30.58 ± 1.48 ^b,c^	29.27 ± 0.56 ^c^	28.92 ± 0.73 ^c^	37.23 ± 0.96 ^a^	25.95 ± 0.08 ^d^	32.48 ± 0.98 ^b^
Kaempferol-dihexosyl acetate	10.52 ± 0.50 ^c,d^	11.33 ± 0.05 ^c^	11.04 ± 0.30 ^c,d^	10.41 ± 0.19 ^d^	15.19 ± 0.38 ^a^	8.99 ± 0.22 ^e^	12.74 ± 0.17 ^b^
Kaempferol 3-O-(6″-O-acetyl) glucoside-7-O-rhamnoside	<LOQ	<LOQ	<LOQ	<LOQ	<LOQ	<LOQ	<LOQ
Kaempferol 3-apiosyl-(1->4)-rhamnoside-7-rhamnoside	2.50 ± 0.08 ^d,e^	2.81 ± 0.11 ^c^	2.67 ± 0.13 ^c,d^	2.13 ± 0.10 ^f^	4.79 ± 0.15 ^a^	2.24 ± 0.08 ^e,f^	3.15 ± 0.12 ^b^
Kaempferol 3-O-sinapoyl-caffeoyl-sophoroside 7-O-glucoside isomer I	5.80 ± 0.06 ^b^	5.72 ± 0.21 ^b^	4.61 ± 0.20 ^c^	4.63 ± 0.18 ^c^	8.97 ± 0.23 ^a^	4.88 ± 0.12 ^c^	6.25 ± 0.28 ^b^
Kaempferol 3-O-sinapoyl-caffeoyl-sophoroside 7-O-glucoside isomer II	4.17 ± 0.07 ^c^	3.59 ± 0.15 ^d^	3.11 ± 0.13 ^f^	3.47 ± 0.10 d,^e^	6.45 ± 0.09 ^a^	3.13 ± 0.10 ^e,f^	4.71 ± 0.22 ^b^
3′,4′-Didemethylnobiletin	<LOQ	<LOQ	<LOQ	<LOQ	<LOQ	<LOQ	<LOQ

LOQ: Limit of quantification. Different letters (a–f) in the same line indicate significant differences (*p* < 0.05).

## Data Availability

The original contributions presented in the study are included in the article, further inquiries can be directed to the corresponding author.

## References

[1] Statista. https://www.statista.com/.

[2] Razola-Díaz M.d.C., Guerra-Hernández E.J., Rodríguez-Pérez C., Gómez-Caravaca A.M., García-Villanova B., Verardo V. (2021). Optimization of Ultrasound-Assisted Extraction via Sonotrode of Phenolic Compounds from Orange By-Products. Foods.

[3] Singh B., Singh J.P., Kaur A., Singh N. (2020). Phenolic composition, antioxidant potential and health benefits of citrus peel. Food Res. Int..

[4] Rastogi Y.R., Thakur R., Thakur P., Mittal A., Chakrabarti S., Siwal S.S., Thakur V.K., Saini R.V., Saini A.K. (2022). Food fermentation—Significance to public health and sustainability challenges of modern diet and food systems. Int. J. Food Microbiol..

[5] Aravantinos-Zafiris G., Tzia C., Oreopoulou V., Thomopoulos C.D. (1994). Fermentation of orange processing wastes for citric acid production. J. Sci. Food Agric..

[6] Rivas B., Torrado A., Torre P., Converti A., Domínguez J.M. (2008). Submerged citric acid fermentation on orange peel autohydrolysate. J. Agric. Food Chem..

[7] Leh D.S., Biz A., de Paula D.H.F., Richard P., Gonçalves A.G., Noseda M.D., Mitchell D.A., Krieger N. (2017). Conversion of citric pectin into D-galacturonic acid with high substrate loading using a fermented solid with pectinolytic activity. Biocatal. Agric. Biotechnol..

[8] Kuivanen J., Dantas H., Mojzita D., Mallmann E., Biz A., Krieger N., Mitchell D., Richard P. (2014). Conversion of orange peel to L-galactonic acid in a consolidated process using engineered strains of *Aspergillus niger*. AMB Express.

[9] Li Q., Siles J.A., Thompson I.P. (2010). Succinic acid production from orange peel and wheat straw by batch fermentations of Fibrobacter succinogenes S85. Appl. Microbiol. Biotechnol..

[10] Gaind S. (2017). Exploitation of Orange Peel for Fungal Solubilization of Rock Phosphate by Solid State Fermentation. Waste Biomass Valorization.

[11] Yalemtesfa B., Alemu T., Santhanam A. (2010). Solid substrate fermentation and conversion of orange waste in to fungal biomass using *Aspergillus niger* KA-06 and *Chaetomium* Spp KC-06. Afr. J. Microbiol. Res..

[12] Ahmadi F., Zamiri M.J., Khorvash M., Banihashemi Z., Bayat A.R. (2015). Chemical composition and protein enrichment of orange peels and sugar beet pulp after fermentation by two Trichoderma species. Iran. J. Vet. Res..

[13] Mantzouridou F.T., Paraskevopoulou A., Lalou S. (2015). Yeast flavour production by solid state fermentation of orange peel waste. Biochem. Eng. J..

[14] Kantifedaki A., Kachrimanidou V., Mallouchos A., Papanikolaou S., Koutinas A.A. (2018). Orange processing waste valorisation for the production of bio-based pigments using the fungal strains *Monascus purpureus* and *Penicillium purpurogenum*. J. Clean. Prod..

[15] Sepúlveda L., Laredo-Alcalá E., Buenrostro-Figueroa J.J., Ascacio-Valdés J.A., Genisheva Z., Aguilar C., Teixeira J. (2020). Ellagic acid production using polyphenols from orange peel waste by submerged fermentation. Electron. J. Biotechnol..

[16] Ahmed N.E., Awad H.M. (2021). Optimizing the production of pectinase of orange peel waste by penicillium chrysogenum mf318506 using response surface methodology in submerged fermentation. J. Microbiol. Biotechnol. Food Sci..

[17] Wang Y., Wu J., Lv M., Shao Z., Hungwe M., Wang J., Bai X., Xie J., Wang Y., Geng W. (2021). Metabolism Characteristics of Lactic Acid Bacteria and the Expanding Applications in Food Industry. Front. Bioeng. Biotechnol..

[18] de la Torre I., Ladero M., Santos V.E. (2020). d-lactic acid production from orange waste enzymatic hydrolysates with *L. delbrueckii* cells in growing and resting state. Ind. Crops Prod..

[19] Drouault S., Corthier G. (2001). Health effects of lactic acid bacteria ingested in fermented milk. Vet. Res..

[20] Signorini M., Salazar J.A., Ponce-Alquicira E., Guerrero-Legarreta I. (2007). Effect of lactic acid and lactic acid bacteria treatment on myofibrillar protein degradation and dynamic rheology of beef. J. Texture Stud..

[21] Verni M., De Mastro G., De Cillis F., Gobbetti M., Rizzello C.G. (2019). Lactic acid bacteria fermentation to exploit the nutritional potential of Mediterranean faba bean local biotypes. Food Res. Int..

[22] Bergillos-Meca T., Cabrera-Vique C., Artacho R., Moreno-Montoro M., Navarro-Alarcón M., Olalla M., Giménez R., Seiquer I., Ruiz-López M.D. (2015). Does Lactobacillus plantarum or ultrafiltration process improve Ca, Mg, Zn and P bioavailability from fermented goats’ milk?. Food Chem..

[23] Curiel J.A., Pinto D., Marzani B., Filannino P., Farris G.A., Gobbetti M., Rizzello C.G. (2015). Lactic acid fermentation as a tool to enhance the antioxidant properties of *Myrtus communis* berries. Microb. Cell Fact..

[24] Pontonio E., Montemurro M., Pinto D., Marzani B., Trani A., Ferrara G., Mazzeo A., Gobbetti M., Rizzello C.G. (2019). Lactic acid fermentation of pomegranate juice as a tool to improve antioxidant activity. Front. Microbiol..

[25] Muñoz R., de las Rivas B., López de Felipe F., Reverón I., Santamaría L., Esteban-Torres M., Curiel J.A., Rodríguez H., Landete J.M., Frias J., Martinez-Villaluenga C., Peñas E. (2017). Chapter 4—Biotransformation of Phenolics by Lactobacillus plantarum in Fermented Foods. Fermented Foods in Health and Disease Prevention.

[26] Mathur H., Beresford T.P., Cotter P.D. (2020). Health Benefits of Lactic Acid Bacteria (LAB) Fermentates. Nutrients.

[27] Zhang H., Cai Y. (2014). Lactic Acid Bacteria: Fundamentals and Practice.

[28] Huang J.Y., Huang M.L., Kao C.Y., Fang T.J. (2017). Orange peel fiber and Tremella flava fermented powder effectively induce exopolysaccharide production by *Lactobacillus plantarum* SLC 13. Int. J. Agric. Biol..

[29] Ricci A., Díaz A.B., Lazzi C., Blandino Garrido A.M. (2023). Valorization of orange peels exploiting fungal solid-state and lacto-fermentation. J. Sci. Food Agric..

[30] Razola-Díaz M.d.C., Verardo V., Gómez-Caravaca A.M., García-Villanova B., Guerra-Hernández E.J. (2023). Mathematical Modelling of Convective Drying of Orange By-Product and Its Influence on Phenolic Compounds and Ascorbic Acid Content, and Its Antioxidant Activity. Foods.

[31] De Montijo-Prieto S., Castro D.J., Reina J.C., Jimenez-Valera M., Ruiz-Bravo A. (2019). Draft genome sequence of Lactobacillus plantarum C4 (CECT 9567), a potential probiotic strain isolated from kefir. Arch. Microbiol..

[32] De Montijo-Prieto S., Razola-Díaz M.d.C., Barbieri F., Tabanelli G., Gardini F., Jiménez-Valera M., Ruiz-Bravo A., Verardo V., Gómez-Caravaca A.M. (2023). Impact of Lactic Acid Bacteria Fermentation on Phenolic Compounds and Antioxidant Activity of Avocado Leaf Extracts. Antioxidants.

[33] Razola-Díaz M.d.C., De Montijo-Prieto S., Aznar-Ramos M.J., Jiménez-Valera M., Ruiz-Bravo A., Verardo V., Gómez-Caravaca A.M. (2023). Effect of Lactic Acid Bacteria Fermentation on the Polar Compounds Content with Antioxidant and Antidiabetic Activity of Avocado Seed Extracts. Fermentation.

[34] Verni M., Pontonio E., Krona A., Jacob S., Pinto D., Rinaldi F., Verardo V., Díaz-de-Cerio E., Coda R., Rizzello C.G. (2020). Bioprocessing of Brewers’ Spent Grain Enhances Its Antioxidant Activity: Characterization of Phenolic Compounds and Bioactive Peptides. Front. Microbiol..

[35] Brand-Williams W., Cuvelier M.E., Berset C. (1995). Use of a free radical method to evaluate antioxidant activity. LWT Food Sci. Technol..

[36] Re R., Pellegrini N., Proteggente A., Pannala A., Yang M., Rice-Evans C. (1999). Antioxidant activity applying an improved ABTS radical cation decolorization assay. Free Radic. Biol. Med..

[37] Ricci A., Diaz A.B., Caro I., Bernini V., Galaverna G., Lazzi C., Blandino A. (2019). Orange peels: From by-product to resource through lactic acid fermentation. J. Sci. Food Agric..

[38] Fritsch C., Jänsch A., Ehrmann M.A., Toelstede S., Vogel R.F. (2017). Characterization of Cinnamoyl Esterases from Different Lactobacilli and Bifidobacteria. Curr. Microbiol..

[39] Muñoz R., de Las Rivas B., Curiel J.A., Rodríguez H., Esteban-Torres M., Reverón I., Santamaría L., Landete J.M., Plaza-Vinuesa L., Sánchez-Arroyo A. (2024). Food phenolics and *Lactiplantibacillus plantarum*. Int. J. Food Microbiol..

[40] Sánchez-Maldonado A.F., Schieber A., Gänzle M.G. (2011). Structure-function relationships of the antibacterial activity of phenolic acids and their metabolism by lactic acid bacteria. J. Appl. Microbiol..

[41] Filannino P., Bai Y., Di Cagno R., Gobbetti M., Gänzle M.G. (2015). Metabolism of phenolic compounds by *Lactobacillus* spp. during fermentation of cherry juice and broccoli puree. Food Microbiol..

[42] Rozès N., Peres C. (1998). Effects of phenolic compounds on the growth and the fatty acid composition of *Lactobacillus plantarum*. Appl. Microbiol. Biotechnol..

[43] Filannino P., Di Cagno R., Gobbetti M. (2018). Metabolic and functional paths of lactic acid bacteria in plant foods: Get out of the labyrinth. Curr. Opin. Biotechnol..

[44] Esteban-Torres M., Landete J.M., Reverón I., Santamaría L., de las Rivas B., Muñoz R. (2015). A *Lactobacillus plantarum* esterase active on a broad range of phenolic esters. Appl. Environ. Microbiol..

[45] Soumya M.P., Nampoothiri K.M. (2021). An overview of functional genomics and relevance of glycosyltransferases in exopolysaccharide production by lactic acid bacteria. Int. J. Biol. Macromol..

[46] Xu L., Qi T., Xu L., Lu L., Xiao M. (2016). Recent progress in the enzymatic glycosylation of phenolic compounds. J. Carbohydr. Chem..

[47] Feng J., Zhang P., Cui Y., Li K., Qiao X., Zhang Y.T., Li S.M., Cox R.J., Wu B., Ye M. (2017). Regio- and Stereospecific O-Glycosylation of Phenolic Compounds Catalyzed by a Fungal Glycosyltransferase from *Mucor hiemalis*. Adv. Synth. Catal..

[48] Xie K., Dou X., Chen R., Chen D., Fang C., Xiao Z., Dai J. (2017). Two novel fungal phenolic UDP glycosyltransferases from *Absidia coerulea* and *Rhizopus japonicus*. Appl. Environ. Microbiol..

[49] Xie L., Zhang L., Bai J., Yue Q., Zhang M., Li J., Wang C., Xu Y. (2019). Methylglucosylation of Phenolic Compounds by Fungal Glycosyltransferase-Methyltransferase Functional Modules. J. Agric. Food Chem..

[50] Kralj S., Van Geel-Schutten G.H., Rahaoui H., Leer R.J., Faber E.J., Van der Maarel M.J.E.C., Dijkhuizen L. (2002). Molecular Characterization of a Novel Glucosyltransferase from *Lactobacillus reuteri* Strain 121 Synthesizing a Unique, Highly Branched Glucan with α-(1→4) and α-(1→6) Glucosidic Bonds. Appl. Environ. Microbiol..

[51] Lim E.K., Higgins G.S., Li Y., Bowles D.J. (2003). Regioselectivity of glucosylation of caffeic acid by a UDP-glucose:glucosyltransferase is maintained in planta. Biochem. J..

[52] Nishimura T., Kometani T., Takii H., Terada Y., Okada S. (1995). Glucosylation of caffeic acid with *Bacillus subtilis* X-23 α-amylase and a description of the glucosides. J. Ferment. Bioeng..

[53] Kang J., Kim Y.M., Kim N., Kim D.W., Nam S.H., Kim D. (2009). Synthesis and characterization of hydroquinone fructoside using *Leuconostoc mesenteroides* levansucrase. Appl. Microbiol. Biotechnol..

[54] Lu L., Guo Y., Xu L., Qi T., Jin L., Xu L., Xiao M. (2015). Galactosylation of caffeic acid by an engineered β-galactosidase. Drug Discov. Ther..

[55] Johnson J.B., Mani J.S., Broszczak D., Prasad S.S., Ekanayake C.P., Strappe P., Valeris P., Naiker M. (2021). Hitting the sweet spot: A systematic review of the bioactivity and health benefits of phenolic glycosides from medicinally used plants. Phytother. Res..

[56] Mallampalli V., Owens D.K., Kumar D. (2009). Expression and Biochemical Function of Putative Flavonoid GT Clones from Grapefruit and Identification of New Clones using the harvEST Database. Master’s Thesis.

[57] Guo X., Guo A., Li E. (2021). Biotransformation of two citrus flavanones by lactic acid bacteria in chemical defined medium. Bioprocess Biosyst. Eng..

[58] Deba-Rementeria S., Paz A., Estrada O., Vázquez-Araújo L. (2023). Consumer perception and physicochemical characterization of a new product made from lactic acid fermented orange peels. Int. J. Gastron. Food Sci..

[59] Razola-Díaz M.d.C., Sevenich R., Rossi Ribeiro L., Guerra-Hernández E.-J., Schlüter O., Verardo V. (2024). Combined effect of pulse electric field and probe ultrasound technologies for obtaining phenolic compounds from orange by-product. LWT.

[60] Rozan M., Alamri E., Bayomy H. (2022). Fermented Hass avocado kernel: Nutritional properties and use in the manufacture of biscuits. Saudi J. Biol. Sci..

[61] Li Z., Teng J., Lyu Y., Hu X., Zhao Y., Wang M. (2019). Enhanced Antioxidant Activity for Apple Juice Fermented with *Lactobacillus plantarum* ATCC14917. Molecules.

[62] Jin Y., Wu J., Hu D., Li J., Zhu W., Yuan L., Chen X., Yao J. (2023). Gamma-Aminobutyric Acid-Producing *Levilactobacillus brevis* Strains as Probiotics in Litchi Juice Fermentation. Foods.

[63] Gong L., Li T., Feng J., Yin J., Zou X., Wang J., Wang B. (2023). Enhanced DPPH Radical Scavenging Activity and Enriched γ-Aminobutyric Acid in Mulberry Juice Fermented by the Probiotic *Lactobacillus brevis* S3. Fermentation.

[64] Piazzon A., Vrhovsek U., Masuero D., Mattivi F., Mandoj F., Nardini M. (2012). Antioxidant activity of phenolic acids and their metabolites: Synthesis and antioxidant properties of the sulfate derivatives of ferulic and caffeic acids and of the acyl glucuronide of ferulic acid. J. Agric. Food Chem..

[65] Ben Abdallah M., Chadni M., M’hiri N., Brunissen F., Rokbeni N., Allaf K., Besombes C., Ioannou I., Boudhrioua N. (2023). Intensifying Effect of Instant Controlled Pressure Drop (DIC) Pre-Treatment on Hesperidin Recovery from Orange Byproducts: In Vitro Antioxidant and Antidiabetic Activities of the Extracts. Molecules.

